# Atrial Fibrillation and Hyperthyroidism*

**Published:** 2005-10-01

**Authors:** Jayaprasad N, Johnson Francis

**Affiliations:** Department of Cardiology, Medical College Calicut, Kerala, India

**Keywords:** Atrial fibrillation, hyperthyroidism, embolism

## Abstract

Atrial fibrillation occurs in 10 - 15% of patients with hyperthyroidism. Low serum thyrotropin concentration is an independent risk factor for atrial fibrillation. Thyroid hormone contributes to arrythmogenic activity by altering the electrophysiological characteristics of atrial myocytes by shortening the action potential duration, enhancing automaticity and triggered activity in the pulmonary vein cardio myocytes. Hyperthyroidism results in excess mortality from increased incidence of circulatory diseases and dysrhythmias. Incidence of cerebral embolism is more in hyperthyroid patients with atrial fibrillation, especially in the elderly and anti-coagulation is indicated in them. Treatment of hyperthyroidism results in conversion to sinus rhythm in up to two-third of patients. Beta-blockers reduce left ventricular hypertrophy and atrial and ventricular arrhythmias in patients with hyperthyroidism. Treatment of sub clinical hyperthyroidism is controversial. Optimizing dose of thyroxine treatment in those with replacement therapy and beta-blockers is useful in exogenous subclinical hyperthyroidism.

## Introduction

Atrial fibrillation is the most common cardiac arrhythmia other than sinus tachycardia encountered in hyperthyroidism. Atrial fibrillation occurs in 10-15% of patients with hyperthyroidism [1]. It may be the presenting problem in some of them. Higher prevalence occurs in elderly and in those with other coexisting risk factors for atrial fibrillation. Low serum thyroid stimulating hormone is an independent risk factor for development of atrial fibrillation [2]. Atrial fibrillation in thyrotoxicosis is associated with significant mortality and morbidity resulting from embolic events [3].

Thyroid hormones exert their cardiovascular effects either directly through nuclear thyroid receptors or indirectly by influencing sympathoadrenergic system and altering peripheral vascular resistance. Binding of thyroid hormones to nuclear receptors result in increased gene transcription of cardiac myocyte proteins [4]. Thyroid hormones upregulate sarcoplasmic Calcium ATPase, myosin heavy chain alfa, voltage gated K+ channels, Na+ channels and beta1 adrenergic receptors [5-8]. These effects result in increased heart rate, systolic hypertension, increased ventricular contractility and cardiac hypertrophy. Changes in electrophysiological characteristics of atria result in dysrhythmias, especially atrial fibrillation, in patients with hyperthyroidism [9]. Thyroid hormones reduce peripheral vascular resistance [10] and increase oxygen demand of tissues, thus increasing cardiac workload.

## Epidemiology

Sinus tachycardia is the most common arrhythmia in hyperthyroidism [11]. Atrial fibrillation is reported in 10 - 15% of patients with hyperthyroidism. Prevalence increases with age. In the study by Agner T et al, 25% of hyperthyroid patients older than 60 years had atrial fibrillation compared to 5% in patients less than 60 years of age [12]. Patients with toxic nodular goiter had an increased incidence of atrial fibrillation compared to younger patients with Grave’s disease, probably due to their increased age. (43% versus 10%). Iwasaki T et al reported that 21% of patients with Grave’s disease had atrial fibrillation with significant difference between those above and below 40 years of age (31% versus 0%) [13]. In a large study by Krahn et al, overt hyperthyroidism accounted for <1% of cases of new onset atrial fibrillation. According to these investigators, although serum thyrotropin should be measured in all patients with new onset atrial fibrillation to rule out hyperthyroidism, this association is rather uncommon in the absence of additional symptoms and signs of hyperthyroidism [14]. However, 13% of patients with unexplained atrial fibrillation, had biochemical evidence of hyperthyroidism in another report [2].

Lars Frost et al identified that among 40,628 patients with hyperthyroidism from Danish National Registry over a 20 year period, 8.3% had atrial fibrillation or flutter within 30 days from the date of diagnosis. The risk factors for atrial fibrillation in patients with hyperthyroidism were similar to those in general population like age, male sex, ischemic heart disease, congestive heart failure and valvular heart disease [15].  Hyperthyroidism was associated with excess mortality compared to general population in a cohort of 7209 hyperthyroid subjects, treated with radio iodine. Excess mortality was due to circulatory diseases. Both cardiovascular (Standardized mortality ratio 1.2, 95% CI  1.2 to 1.3; p  <0.001) and cerebrovascular (Standardized mortality ratio 1.4, 95% CI 1.2 to 1.5, p <0.001) mortality rates were high in hyperthyroid subjects. The Standardized mortality ratio for dysrhythmia including atrial fibrillation was 1.8 (95% CI 1.5 to 1.9, p value <0.001) [16]. In another study, 1762 hyperthyroid women treated with radio iodine were followed for more than 14 years and there was increased mortality from cardiovascular disease [17]. Cardiac arrhythmias, of which atrial fibrillation is most frequent contribute to excess mortality from cardiovascular and cerebrovascular events by inducing heart failure and predisposing to embolic events.

## Subclinical Hyperthyroidism and Atrial Fibrillation

Sub clinical hyperthyroidism is defined as low serum thyrotropin concentration in an asymptomatic patient with normal serum T3 and T4 concentration. It has a prevalence of 0.5% to 3.9% in adults [18] and 11.8% in elderly [19].

Auer J et al, studied 23,638 persons and found that atrial fibrillation occurred in 13.8% patients with overt hyperthyroidism and 12.7% patients with sub clinical hyperthyroidism, compared to 2.3% in euthyroid. The prevalence of atrial fibrillation in patients with low serum thyrotropin concentration was 13.3% compared to 2.3% in persons with normal values. The relative risk of atrial fibrillation in subjects with low serum thyrotropin and normal free T3,T4 values compared to those with normal serum thyrotropin was 5.2. Thus low serum thyrotropin concentration  is associated with >5 fold higher likelihood for atrial fibrillation with no significant difference between overt and subclinical hyperthyroidism [20]. Clark T Sawin et al followed 2007 patients from the original cohort of Framingham Heart Study, who were older than 60 years, for 10 years, for development of atrial fibrillation. Subjects with low thyrotropin (<0.1 mU/L) had 28% incidence of atrial fibrillation, compared with 11% in normal subjects. The relative risk for development of atrial fibrillation in patients with low thyrotropin was 3.1 [1]. The subjects who had slightly low serum thyrotropin concentrations (0.1 to 0.4mU/L) also had higher risk than those with normal concentrations (relative risk 1.6; p=0.05)

Subclinical hyperthyroidism can be endogenous as occurring in Grave’s disease, multinodular goiter or autonomous toxic nodules or exogenous due to thyroxine therapy. Exogenous subclinical hyperthyroidism is the most common cause of subclinical hyperthyroidism [21]. Subclinical hyperthyroidism is also associated with increased cardiovascular morbidity and mortality. 1191 subjects aged over 60 years, when followed up for 10 years, those with low serum thyrotropin (<0.5 mU/L) had higher mortality compared to control population. This excess mortality resulted mainly from circulatory diseases and supraventricular arrhythmias including atrial fibrillation also contributed [22].

## Pathogenesis

Effects of thyroid hormones on ion currents of atrial myocytes contribute to genesis of atrial fibrillation. Hyperthyroidism is associated with shortening of action potential duration (APD) resulting in a substrate for atrial fibrillation. A study on the effects of thyroid hormones on mRNA expression and currents of major ionic channels in murine atrium showed that T3 increased expression of the Kv1.5 mRNA and decreased L-type Calcium channel mRNA expression. Action potential duration was shorter in hyperthyroid than in euthyroid myocytes. The ultra-rapid delayed rectifier potassium currents were considerably increased in hyperthyroid than in euthyroid myocytes, whereas the transient outward potassium currents were unchanged. L-type calcium currents were decreased in hyperthyroid than in euthyroid myocytes. T3 increased the outward currents and decreased the inward currents resulting in reduced action potential duration [23]. In another study action potential duration and whole cell currents were studied in myocytes from left and right atria from control and hyperthyroid mice. Hyperthyroidism resulted in more significant APD shortening and greater delayed rectifier potassium current increases in the right atrium than in the left atrium which can contribute to the propensity for atrial arrhythmias [24]. Studies using rabbit pulmonary vein cardiomyocytes have shown that thyroid hormone decreases the APD in pulmonary vein cardiomyocytes which can facilitate the genesis of reentrant circuits. Incubation with thyroid hormone also increased spontaneous activity in the pulmonary vein cardiomyocytes similar to the effect on sinoatrial node cells. Thyroid hormone increased the occurrence of delayed after-depolarisation in pulmonary vein beating and non-beating cardiomyocytes. Incidence of early after-depolarisation was increased in beating cardiomyocytes following incubation with thyroid hormone. Thus thyroid hormone may induce the occurrence of paroxysmal atrial fibrillation through the increase of triggered activity or automaticity in pulmonary veins [25].

ECG may be helpful in identifying hyperthyroid subjects at risk for developing atrial fibrillation. Maximum P wave duration and P wave dispersion were higher in both subclinical and overt hyperthyroidism. P maximum and P wave dispersion were significant predictors of paroxysmal atrial fibrillation [26].

Thyroid hormone potentiates the effect of adrenergic system on heart. Catecholamine levels are either normal or decreased in thyrotoxicosis. Facilitation of action of catecholamines is by increasing tissue sensitivity by increased transcription of beta adrenergic receptors  [27] and structural similarity to catecholamines [28]. Hyperthyroidism is associated with reduced vagal activity and reduced heart rate variability which can persist despite restoration of euthyroidism [29].

## Embolic Events and Anticoagulation

Thyrotoxicosis is complicated by thromboembolism in approximately 15% of cases [3].    In a retrospective study of  610 patients with hyperthyroidism the risk of cerebrovascular events was greater in those with atrial fibrillation. 15%  had atrial fibrillation with the highest frequency in elderly patients. A total of 27 (4.4%) cerebrovascular events occurred, 13% in those having atrial fibrillation and 3% in those with sinus rhythm. Advanced age rather than the presence of atrial fibrillation was the important risk factor for embolism. From this study the indication for prophylactic anticoagulation is doubtful in hyperthyroid patients with atrial fibrillation [30]. In younger patients with hyperthyroidism and atrial fibrillation who do not have other heart disease, hypertension or other risk factors for embolism, the risk of anticoagulant therapy probably outweighs the benefit [31]. The risk of embolism in thyrotoxic atrial fibrillation exceeds that of lone atrial fibrillation. The majority of clinically evident emboli in patients with hyperthyroidism and atrial fibrillation involves central nervous system and occur early in the course of the disease [32]. Elderly patients with thyrotoxicosis and atrial fibrillation and those with other risk factors for thromboembolism have significantly increased risk for arterial thromboembolism and anticoagulant treatment is indicated. Elderly patients are particularly at risk for hemorrhagic complications and hence close monitoring of prothrombin time is required in elderly patients on warfarin. Antiplatelet agents like aspirin may afford some protection against cardioembolic stroke in patients with atrial fibrillation, although these are more effectively prevented by anticoagulation [33].

## Treatment

Mainstay of treatment in patients with atrial fibrillation and hyperthyroidism is restoration of euthyroid status. This is by use of antithyroid drugs like carbimazole, propyl thiouracil or radio-iodine. Surgery of thyroid gland is done after achieving euthyroid status by drugs. Beta blockers like propranalol or atenolol are useful in thyrotoxic atrial fibrillation to reduce heart rate and cardiac failure [34,35]. Restoration of euthyroid status is frequently associated with conversion to sinus rhythm. In a study of 163 patients with thyrotoxicosis and atrial fibrillation, 62% were in sinus rhythm within 8-10 weeks after achieving euthyroid state [36]. After 3 months only few will revert spontaneously to sinus rhythm. Electrical or pharmacologic cardioversion may be attempted in patients remaining in atrial fibrillation after achieving euthyroid status. Rate of reversion to sinus rhythm is less in older patients and in those with longer duration of atrial fibrillation and structural heart disease. In another study, of the 256 patients who underwent surgery for thyrotoxicosis 23% had preoperative atrial fibrillation. After surgery 47% of them reverted to sinus rhythm and the rest had better responsiveness to antiarrhythmic drugs. Restoration of sinus rhythm occurred mostly in patients younger than 50 years while in older patients atrial fibrillation persisted [37].

Treatment of subclinical hyperthyroidism is controversial. Some authors advocate careful follow up of these patients for development of overt hypothyroidism, cardiac dysrhythmias and other circulatory complications [38]. But others have suggested routine treatment for subclinical hyperthyroidism as it is associated with adverse cardiac events. Antithyroid drugs or radio-iodine may be useful especially in those with nodular goiter and cardiac risk factors [39]. Treatment of subclinical hyperthyroidism with antithyroid drugs was shown to reduce left ventricular mass index, heart rate, atrial and ventricular premature beats and atrial fibrillation. Many patients in this group with subclinical hyperthyroidism had symptoms, high Wayne clinical index and echocardiographic abnormalities which reduced with treatment [40]. Biondi et al observed that treatment with selective beta1 blocker bisoprolol reduces left ventricular mass index and atrial arrhythmias in patients taking long term thyrotropin suppressive therapy with thyroxine [41]. Dosage of thyroxine in patients receiving replacement therapy should be adjusted to a normal and not suppressed thyrotropin level [42].

## Conclusion

Atrial fibrillation is a major cause of morbidity and mortality in overt as well as subclinical hyperthyroidism. It is associated with cerebral embolic events, especially in elderly and those with co-morbid risk factors. Treatment with anti-thyroid drugs and beta-blockers is indicated in most of the cases.
